# Haploidentical peripheral blood stem cell transplantation combined with unrelated cord blood in hematologic malignancy patients: A report of 80 cases

**DOI:** 10.3389/fimmu.2022.980464

**Published:** 2022-09-02

**Authors:** Cong Zeng, Yan Chen, Juan Hua, Yi Liu, Ting-ting Cheng, Xia Ma, Xu Chen, Shi-yu Wang, Ya-jing Xu

**Affiliations:** ^1^ Department of Hematology, Xiangya Hospital, Central South University, Changsha, China; ^2^ National Clinical Research Center for Geriatric Diseases (Xiangya Hospital), Changsha, China; ^3^ Hunan Hematologic Neoplasms Clinical Medical Research Center, Xiangya Hospital, Central South University, Changsha, China; ^4^ National Clinical Research Center for Hematologic Diseases, The First Affiliated Hospital of Soochow University, Changsha, China

**Keywords:** peripheral blood, cord blood, haploidentical donor, allogeneic hematopoietic stem cell transplantation, hematologic malignancy

## Abstract

The outcomes of 80 patients with hematologic malignancies who received haploidentical peripheral blood stem cell transplantation (haplo-PBSCT) combined with unrelated cord blood (UCB) from March 2017 to June 2020 were analyzed in this retrospective study. Anti-thymocyte globulin(ATG) was administered at a dose of 7.5 mg/kg. The median time for neutrophil and platelet engraftment was 13(range: 8-22) days and 14(range: 8-103) days, respectively. The 30-day cumulative incidence of neutrophil engraftment was 100%, and the 100-day cumulative incidence of platelet engraftment was 95%. All patients achieved complete haploidentical peripheral blood stem cell engraftment, and no cord blood chimerism was observed. The cumulative incidence of grades II-IV and grades III-IV acute graft-versus-host disease (aGVHD) on 100-day was 26.3%(95%CI: 17.2%–36.3%) and 5.0%(95%CI: 1.6%–11.4%), respectively. The estimated cumulative incidence of chronic GVHD (cGVHD) and moderate-severe cGVHD at 3-year was 43.3%(95%CI: 31.6%–54.4%) and 16.0%(95%CI: 8.7%–25.2%), respectively. The estimated 3-year cumulative incidence of relapse and non-relapse mortality was 18.8%(95%CI: 10.0%–29.7%) and 17.8%(95%CI: 9.9%–27.5%), respectively. The estimated 3-year probabilities of overall survival, disease-free survival, GVHD/relapse-free survival were 77.6%(95%CI: 68.3%–88.1%), 63.4%(95%CI: 52.6%–76.5%), and 55.5%(95%CI: 44.8%–68.7%), respectively. These satisfying results suggested that haplo-PBSCT combined with UCB is an alternative transplantation protocol for hematologic malignancies.

## Introduction

Allogeneic hematopoietic stem cell transplantation (allo-HSCT) is currently the leading therapy for hematologic malignancies. Haploidentical HSCT (haplo-HSCT) is an alternative for patients who require urgent transplantation but do not have a matched sibling donor (MSD). In recent years, the number of haplo-HSCT has increased rapidly worldwide due to the development of T-cell depletion (TCD) protocol (1, 2). Currently, three dominant TCD protocols are used in haplo-HSCT: *in vivo* TCD protocol with posttransplant cyclophosphamide (PTCY), *in vitro* TCD protocol, and *in vivo* TCD protocol with granulocyte colony-stimulating factor (G-CSF) and anti-thymocyte globulin (ATG) (termed as “Beijing protocol”). The PTCY-based haploidentical transplantation protocol is widely used in Europe and USA; it usually uses peripheral blood stem cells (PBSCs) as the graft source. Several studies have compared the efficacy of PB grafts and bone marrow (BM) grafts in haplo-HSCT using PTCY, indicating faster engraftment and higher incidence of graft-versus-host disease (GVHD) in PB compared to BM grafts, but no significant difference was detected in the long-term survival between the two groups (3–5). “Beijing protocol” is a G-CSF/ATG-based haploidentical BM plus PB transplantation protocol was established by Huang et al. The standard ATG dose is 10 mg/kg in “Beijing protocol.” By 2019, “Beijing protocol” accounted for 94% of haplo-HSCT in China (1). A prospective study found comparable outcomes but rapid hematopoietic reconstitution in G-CSF/ATG-based haploidentical peripheral blood stem cell transplantation (haplo-PBSCT) compared to “Beijing protocol;” however, the incidence of GVHD was generally high in this study (6). Nonetheless, only a few studies have explored this protocol, thereby necessitating need multicenter, prospective, randomized trials to confirm the outcomes.

Cord blood is another widely used source of grafts. The incidence of GVHD in cord blood transplantation is low, but the limited cell count results in delayed hematopoietic reconstruction (7). In order to achieve both rapid hematopoietic reconstitution and a low incidence of GVHD, some studies have combined haplo-HSCT with unrelated cord blood (UCB) and found that this protocol provides the advantages of both methods (8–10). However, the source of stem cells in most of these studies was BM plus PB combined with UCB, and only a few studies utilized pure PB combined with UCB. Therefore, we attempted to use UCB to assist haplo-PBSCT, which avoids the risks of general anesthesia and the pain of bone marrow collection in the donor, thereby easing the operation. Also, the dose of ATG was reduced to 7.5 mg/kg due to the addition of UCB. Finally, we analyzed the outcomes of 80 patients with hematologic malignancies who received haplo-PBSCT combined with UCB (haplo-cord-PBSCT) at the Department of Hematology, Xiangya Hospital of Central South University between March 2017 and June 2020.

## Patients and methods

### Patients

A total of 80 patients with hematologic malignancies who received haplo-cord-PBSCT were retrospectively analyzed in this study from March 2017 to June 2020. Patients who met the following inclusion criteria were recruited in this study: 1. Acute leukemia (AL) diagnosis according to World Health Organization (WHO) diagnostic criteria, with risk stratification according to NCCN guidelines; myelodysplastic syndrome (MDS) diagnosis according to FAB classification, with risk stratification referring to the International Prognostic Scoring System (IPSS); refractory relapse patients were defined as high-risk patients. 2. Patients receiving haplo-cord-PBSCT. 3. No severe impairment of the liver, kidney, lung, and cardiac function. All patients voluntarily signed the informed consent forms that were reviewed by our hospital ethics committee.

### Donors and cord blood selection

HLA typing was assessed in both donors and recipients at a high resolution. Before transplantation, the HLA antibodies were tested in all patients, and donor-specific antibodies (DSA) were negative or weakly positive (MFI<4000) in all patients. The weakly-positive patients were not treated with plasmapheresis or rituximab. HLA-A, -B, -C, -DRB1, -DQB1 locus of UCB were detected at a high resolution. The DSA of cord blood was negative. UCB should be selected according to the following criteria: (1) >5/10 HLA-matching locus, and at least one match on each type locus; (2) minimum mononuclear cell (MNC) count >1×10 ^7^/kg after thawing. Blood type matching was preferred if the HLA type of UCB was identical.

### Conditioning regimen

All patients were treated with a modified busulfan (BU)/cyclophosphamide (CY)-based regimen: cytarabine 4 g/m^2^/d, -8 d– -7 d; BU 3.2 mg/kg/d, -6 d– -4 d; CY 1.8 g/m^2^/d, -3d– -2d; simustine 250 mg/m^2^, -2 d; rabbit ATG 2.5 mg/kg/d, -3d– -1d.

### Stem cell source

Haplo-PBSCs were mobilized using G-CSF (7.5–10 μg/kg/d) from -4 d. The number of PBSCs should meet one of the following criteria: (1) MNC cell count >6–8×10^8^/kg (2) CD34^+^ cell count ≥5×10^6^/kg. If the number of mobilized stem cells on the first day is insufficient, mobilization can be continued on the following day for collection. PBSCs were transfused on d1, and UCB was transfused on d2. Then, UCB was transfused >12 h after PBSC transfusion, and the MNC cell count in the transfused UCB was 1×10^7^/kg.

### GVHD prophylaxis and treatment

A triple prophylaxis regimen of cyclosporine A (CsA), short course methotrexate (MTX), and mycophenolate mofetil (MMF) was applied to prevent GVHD. The prophylaxis regimen consisted of the following: CsA (2.5mg/kg/d) was administered intravenously from -9d, and the blood level of CsA was regularly monitored to maintain the minimum blood level range of 200–250 μg L until the patient gradually moved to oral administration; MTX at specific dosages (15 mg/m^2^, +1d; 10 mg/m^2^, +3d, +6d, and +11d); MMF was administered orally at a dosage of 0.5 g twice daily from -9d, halved at +30d, and discontinued at +45d to +60d if GVHD does not occur. Intravenous administration of 2 mg/kg/d methylprednisolone is chosen for GVHD of grade II or higher, followed by a second-line regimen such as CD25 monoclonal antibody for patients who do not respond well to methylprednisolone.

### Supportive care and post-transplantation monitoring

Intestinal preparation before transplantation was performed with gentamicin and nystatin administration. Prophylactic antibiotics and antifungals and antivirals were given at the start of the conditioning regimen. After transplantation, bone marrow examinations were performed at least monthly during the first 6 months to assess donor chimerism and relapse. Bone marrow was monitored every 1–1.5 months during the second half-year after transplantation and every 1.5–2 months during the first year post-transplantation.

### Definition

Neutrophil engraftment was defined as ANC >0.5×10^9^/L for 3 successive days. Platelet engraftment was defined as PLT >20×10^9^/L for 7 successive days and independent of platelet transfusion. Donor-recipient chimerism >95% was considered a fully donor chimerism. Minimal residual disease(MRD) was defined as the number of leukemic or malignant cells remaining in the patient’s body below the sensitivity of morphological detection when the clearance effect was not achieved after treatment. multiparameter flow cytometry (MFC) was used to detect MRD with the sensitivity of 10^-5^ on bone marrow aspirate samples. Platelet engraftment obtained after 30 days was considered a delayed platelet recovery. Relapse was defined as leukemia cells in peripheral blood or bone marrow >5% or the presence of extramedullary invasion. Deaths due to any cause without recurrence or disease progression were categorized as non-relapse mortality (NRM). Survival in sustained complete remission and without relapse was considered disease-free survival (DFS). GVHD/relapse-free survival (GRFS) was defined as survival without grades III-IV aGVHD and cGVHD requiring systemic therapy, relapse, or disease progression. aGVHD was diagnosed and graded according to the modified Keystone criteria (11). The diagnosis and grading of cGVHD were based on the NIH consensus (12).

### Statistical analysis

The deadline for follow-up was January 31, 2022. The primary endpoint was the cumulative incidences of aGVHD. The secondary endpoint was the engraftment, NRM, relapse, cGVHD, overall survival (OS), DFS and GRFS. The normality was assessed on the measurement data; normally distributed data were expressed as $\bar{\chi }$ ±*s*, and non-normally distributed data were expressed as median (range). Statistical data are expressed as examples (%). Overall survival (OS), DFS, and GRFS were estimated using the Kaplan–Meier method. A competing risk model was used to calculate the cumulative incidences of engraftment, GVHD, NRM, and relapse. Graft failure, relapse, and death due to any cause were considered competing risks for GVHD. Death due to any cause was considered a competing risk for engraftment and relapse. For TRM, relapse was considered a competing risk. P<0.05 indicated a statistically significant difference. Survival analysis was performed using the SPSS26.0 software package (SPSS, Chicago, IL, USA), and competing risk analysis was calculated by R software (version 4.1.1) (https://www.r-project.org/).

## Results

### Patient and donor characteristics

A total of 80 patients were included in this study. The study comprised 42 males and 38 female patients. 38/80 patients had ALL, of which 14 were standard-risk ALL and 24 were high risk ALL, and 42/80 patients had AML/MDS, of which 18 were intermediate-risk and 24 were high-risk. The median follow-up time was 22.4 (range: 2.5–57.8) months. The details are summarized in **Table 1**.

**Table 1:** Characteristics of patient and donor.

Characteristics	N=80
Median age, year (range)	28 (14-56)
Gender, n (%)	
Male	42 (52.50)
Female	38 (47.50)
Disease, n (%)	
AML	37 (46.25)
ALL	38 (47.50)
MDS	5 (6.25)
Risk stratification, n (%)	
AML/MDS	
Intermediate	18 (42.86)
High	24 (57.14)
ALL	
Standard	14 (36.84)
High	24 (63.16)
Median time from diagnosis to HSCT, months (range)	6.0 (0.3-36.0)
Median follow-up time, months (range)	22.4 (2.5-57.8)
Disease status, n (%)	
AML/MDS	
CR1	26 (61.90)
CR≥2	13 (30.95)
Non-CR	3 (7.15)
ALL	
CR1	23 (60.53)
CR≥2	14 (36.84)
Non-CR	1 (2.63)
MRD	
Negative	73 (96.25)
Positive	7 (3.75)
Donor-recipient sex match, n (%)	
Male-male	29 (36.25)
Male-female	26 (32.50)
Female-male	16 (20.00)
Female-female	9 (11.25)
Donor-recipient blood match, n (%)	
Matched	53 (66.25)
Major mismatched	13 (16.25)
Minor mismatched	14 (17.50)
HLA compatibility of, n (%)	
PB	
5/10	45 (56.25)
6/10	23 (28.75)
7/10	5 (6.25)
8/10	5 (6.25)
9/10	2 (2.50)
UCB	
6/10	7 (8.75)
7/10	17 (21.25)
8/10	32 (41.25)
9/10	19 (23.75)
10/10	4 (5.00)
Median PB MNC, ×108/kg (range)	10.50 (4.63-19.30)
Median PB CD34+ cells, ×10 6/kg (range)	5.68 (1.61-14.12)
UCB MNC, ×10 7/kg	1
UCB CD34+ cells, ×10 4/kg	3.42 (1.12-13.56)

AML, acute myeloid leukemia; ALL, acute lymphoblastic leukemia; MDS, myelodysplastic syndrome; HSCT, hemopoietic stem cell transplantation; CR, complete remission; PB, peripheral blood; UCB, unrelated cord-blood.

### Engraftment

The median time to neutrophil and platelet engraftment was 13 (range: 8–22) days and 14 (range: 8–103) days, respectively. The 30-day cumulative incidence of neutrophil engraftment was 100%, and the 100-day cumulative incidence of platelet engraftment was 95%. In this study, 5 patients had delayed platelet recovery. All patients attained full donor chimerism 30 days after transplantation. All patients achieved complete haplo-PBSCT engraftment, and there was no cord blood chimerism.

### GVHD

**Figure 1:**
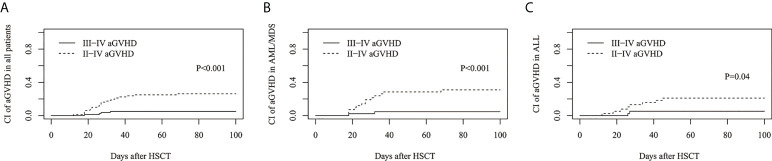
Cumulative incidence (CI) of acute graft-versus-host disease (aGVHD). CI of II-IV aGVHD and III-IV aGVHD in **(A)** all patients, **(B)** AML/MDS and **(C)** ALL.

The cumulative incidence of grades II–IV and grades III-IV aGVHD on day 100 was 26.3%(95%CI: 17.2%–36.3%) and 5.0%(95%CI: 1.6%–11.4%) for all patients, respectively (**Figure 1A**), 30.9%(95%CI: 17.7%–45.2%) and 4.8%(95%CI: 0.8%–14.3%) for AML/MDS patients, respectively (**Figure 1B**), and 21.1%(95%CI: 9.7%–35.2%) and 5.2%(95%CI: 0.9%–15.7%) for ALL patients, respectively (**Figure 1C**).

**Figure 2:**
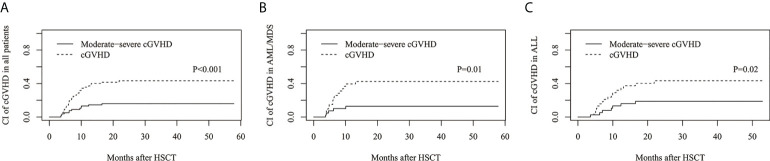
The estimated CI of chronic GVHD (cGVHD). CI of cGVHD and moderate-severe cGVHD in **(A)** all patients, **(B)** AML/MDS and **(C)** ALL.

The estimated cumulative incidence of cGVHD and moderate-severe cGVHD at 3 years was 43.3%(95%CI: 31.6%–54.4%) and 16.0%(95%CI: 8.7%–25.2%), respectively (**Figure 2A**), 42.6%(95%CI: 26.2%–58.0%) and 12.8%(95%CI: 4.6%–25.5%) for AML/MDS patients, respectively (**Figure 2B**), and 43.4%(95%CI: 26.8%–58.9%) and 18.8%(95%CI: 8.1%–32.9%) for ALL patients, respectively (**Figure 2C**).

### Relapse and NRM

**Figure 3:**
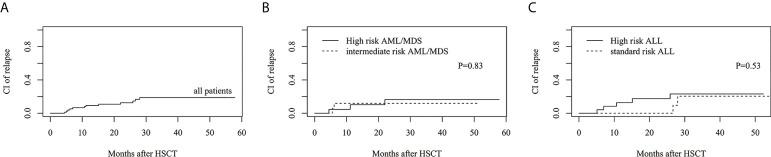
The estimated CI of relapse (CIR). **(A)** CIR in all patients. **(B)** CIR between intermediate risk AML/MDS and high risk AML/MDS. **(C)** CIR between standard risk ALL and high risk ALL.

The estimated 3-year cumulative incidence of relapse (CIR) in all patients was 18.8%(95%CI: 10.0%–29.7%) (**Figure 3A**). The estimated CIR of intermediate- and high-risk AML/MDS was 11.8%(95%CI: 1.8%–31.9%) and 16.5%(95%CI: 3.7%–37.3%), respectively (**Figure 3B**). The estimated CIR of standard- and high-risk ALL was 20.4%(95%CI: 2.5%–50.1%) and 23.1%(95%CI: 7.8%–43.2%), respectively (**Figure 3C**).

**Figure 4:**
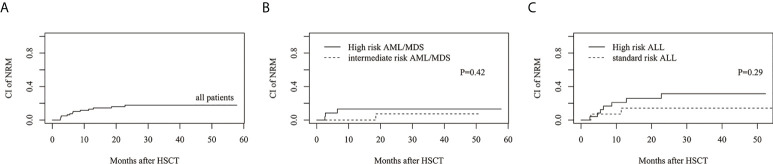
The estimated CI of non-relapse mortality (NRM). **(A)** NRM in all patients. **(B)** NRM between intermediate risk AML/MDS and high risk AML/MDS. **(C)** NRM between standard risk ALL and high risk ALL.

The estimated 3-year cumulative incidence of NRM in all patients was 17.8%(95%CI: 9.9%–27.5%) (**Figure 4A**). The estimated NRM of intermediate and high risk AML/MDS was 7.3 ± 0.5%(95%CI: 0.3%–29.3%) and 13.2%(95%CI: 3.1%–30.5%), respectively (**Figure 4B**). The estimated NRM of standard and high risk ALL was 14.3%(95%CI: 2.1%–37.5%) and 31.5%(95%CI: 13.2%–51.6%), respectively (**Figure 4C**).

### Survival

**Figure 5:**
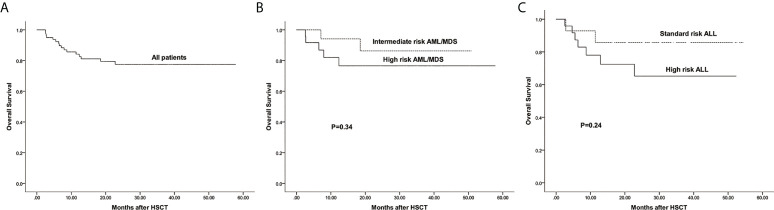
The estimated probability of overall survival (OS). **(A)** OS in all patients. **(B)** OS between intermediate risk AML/MDS and high risk AML/MDS. **(C)** OS between standard risk ALL and high risk ALL.

The estimated 3-year probability of OS in all patients was 77.6%(95%CI: 68.3%–88.1%) (**Figure 5A**). The estimated OS of intermediate and high risk AML/MDS was 86.3%(95%CI: 70.1%–99.5%) and 76.5%(95%CI: 60.3%–97.1%), respectively (**Figure 5B**). The estimated OS of standard and high risk ALL was 85.7%%(95%CI: 69.2%–99.3%) and 65.2%(95%CI: 46.7%–91.1%), respectively (**Figure 5C**).

**Figure 6:**
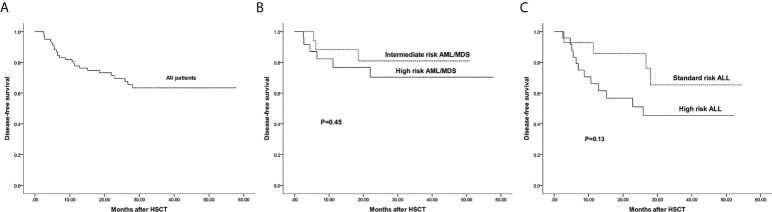
The estimated probability of disease free survival (DFS). **(A)** DFS in all patients. **(B)** DFS between intermediate risk AML/MDS and high risk AML/MDS. **(C)** DFS between standard risk ALL and high risk ALL.

The estimated 3-year probability of DFS in all patients was 63.4%(95%CI: 52.6%–76.5%) (**Figure 6A**). The estimated DFS of intermediate and high risk AML/MDS was 80.9%(95%CI: 63.4%–99.1%) and 70.4%(95%CI: 52.6%–94.1%), respectively (**Figure 6B**). The estimated DFS of standard and high risk ALL was 65.3%(95%CI: 42.2%–99.0%) and 45.4%(95%CI: 28.2%–72.9%), respectively (**Figure 6C**).

**Figure 7:**
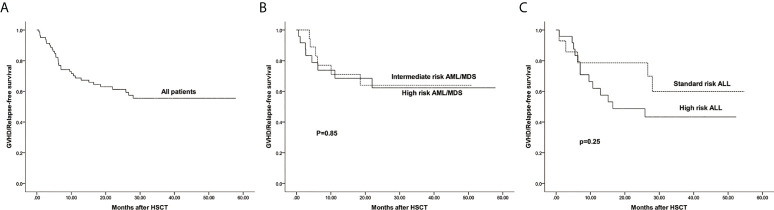
The estimated probability of GVHD/Relapse free survival (GRFS). **(A)** GRFS in all patients. **(B)** GRFS between intermediate risk AML/MDS and high risk AML/MDS. **(C)** GRFS between standard risk ALL and high risk ALL.

The estimated 3-year probability of GRFS in all patients was 55.5%(95%CI: 44.8%–68.7%) (**Figure 7A**). The estimated GRFS of intermediate and high risk AML/MDS was 64.0%(95%CI: 44.4%–92.2%) and 62.3%(95%CI: 44.3%–87.6%), respectively (**Figure 7** **B**). The estimated GRFS of standard and high risk ALL was 59.9%(95%CI: 37.5%–95.7%) and 43.3%(95%CI: 26.8%–69.8%), respectively (**Figure 7C**).

## Discussion

In this study, we reported the outcomes of haplo-cord-PBSCT for the treatment of hematologic malignancies. Due to the addition of UCB, haploidentical peripheral blood transplantation can also achieve a low incidence of GVHD, even when the dose of ATG is reduced to 7.5 mg/kg. Moreover, patients can achieve relatively fast hematopoietic recovery and improved GRFS. Therefore, the current study provided an alternative transplantation option for patients with hematologic malignancies.

Herein, the median time for neutrophil and platelet engraftment was 13 and 14 days, respectively, and the neutrophil and platelet engraftment was 100% and 95%. The Chinese Bone Marrow Transplantation Registration (CBMTR) (13) reported the results of G-CSF/ATG-based haploidentical BM plus PB transplantation; the median time to neutrophil and platelet engraftment was 12 and 17 days, respectively. The neutrophil and platelet engraftment was 96.6% and 94.2%, respectively. In terms of figures alone, our haplo-cord-PBSCT exhibited relatively rapid platelet engraftment and a high rate of engraftment. A meta-analysis study revealed that multiple haplo-HSCT using the PTCY regime achieved a significantly higher engraftment rate in the PB graft than BM graft (14). This was similar to our results; UCB addition may further accelerate engraftment. A rapid engraftment in PB may be associated with higher levels of CD34+ cells than BM. Lyu et al. (15) observed mixed cord blood chimerism in 5.3% of patients in a study of third-party cord blood-assisted haplo-HSCT. However, all our cases achieved complete haploidentical peripheral blood stem cell engraftment, and no cord blood chimerism was detected. This outcome may be associated with a low MNC cell count in the transfused UCB. In the current study, all the MNC cell count of the transfused UCB was 1×10^7^/kg and CD34+ cells was 3.42×104/kg. It could also be attributed to an interval of more than 12h after the end of the PBSC transfusion before the UCB is transfused. Competition between grafts of different origin is common after double UCB transplantation, resulting in the elimination of one graft. One possible explanation is an immune graft-versus-graft effect. So, the absence of T-cell depletion in haplo-cord-HSCT may also attribute to this result.

Typically, PBSCT results in a high incidence of GVHD (16). However, high GVHD was not observed in our study, and the cumulative incidence of II–IV and III-IV aGVHD was 26.3% and 5%, respectively, and cGVHD and moderate-severe cGVHD was 43.3% and 16.0%, respectively. CBMTR reported the cumulative incidence of II-IV and III-IV aGVHD was 26.7% and 8%, respectively and cGVHD was 42.3% (13). Although we reduced the dose of ATG to 7.5 mg/kg and used PBSC as the graft source, the incidence of GVHD was similar to that of haploidentical BM plus PB transplantation with a standard dose of ATG, suggesting a critical role of UCB. Ma et al. (17) evaluated the outcomes of haplo-PBSCT with the standard dose of ATG, and the cumulative incidence of II–IV and III-IV aGVHD was 30% and 7.5%, respectively, and cGVHD and moderate-severe cGVHD at 1-year was 54.9% and 17.4%, respectively. Based on these data, the incidence of GVHD in haplo-PBSCT was relatively higher even with standard doses of ATG without combined UCB, further suggesting that a key role of UCB in reducing the occurrence of GVHD. Ke et al. (9) suggested that UCB might act as an immunomodulator to regulate the hematopoietic microenvironment. Treg cells play a critical role in modulating immune tolerance after transplantation. Mouse experiments indicated that Treg cells significantly reduce the incidence of GVHD by inducing immune tolerance (18). Another meta-analysis clarified that high Treg cell levels in the graft were associated with low GVHD incidence (19). Treg cells are a major component of the immune system in the fetus, and although declined to adult levels at birth, they still have more immunomodulatory effects than peripheral blood (20). In a mouse model of GVHD, studies have improved GVHD scores by prophylactic injection of third-party cord blood-derived, *in vitro* expanded Treg cells and recommended phase I trials of prophylactic infusion of third-party Treg cells in double cord blood transplantation (21). It has been reported that compared with PB there are abundant naïve Treg cells in UCB (22). In this study, however, we did not measure naïve Treg cell levels in cord blood. Next, we intend to conduct prospective studies to reveal the mechanism of cord blood. Although our data suggested a crucial role of UCB in the prevention of GVHD, the mechanism of UCB assistance is not yet fully understood, necessitating additional prospective studies and animal experiments to confirm the findings.

In a multicenter randomized controlled study of two ATG doses (7.5mg/kg vs 10mg/kg) of haploidentical BM plus PB transplantation for hematologic malignancies, the 1-year CI of EBV DNAemia was 20.7% and 40.0% in the 7.5 mg/kg and 10.0 mg/kg groups, respectively (P< 0.001), the 1-year CI of CMV DNAemia was 73.4% and 83.4%, respectively (P = 0.038) (23). The results indicated that ATG at 7.5mg/kg was associated with a lower risk of EBV and CMV infection compared to 10.0 mg/kg. However, unlike other hospitals that used plasma as testing sample, our hospital used whole blood. So we didn’t collect data on this and compare them with other hospitals. Next, we plan to do a matching study to confirm this further.

In this study, the estimated 3-year CIR and NRM were 18.8% and 17.8%, respectively. In the above study comparing the two ATG doses, the 3-year CIR was 17.6% and 15.3%, respectively, and the 3-year NRM was 20.2% and 24.4%, respectively (23). John et al. (24) confirmed the lower relapse of cord blood transplantation than haploidentical transplantation by comparing the clinical outcomes of haploidentical transplantation and cord blood transplantation. The proportion of high-risk patients in our study was relatively high but not relapse was high, suggesting that haplo-cord-PBSCT may have a strong graft-versus-leukemia (GVL) effect; however, this finding needs to be substantiated with a large sample size in a paired randomized controlled trial.

Our data showed that the estimated 3-year OS, DFS, and GRFS were 77.6%, 77.6%, and 55.5%, respectively. In the above study comparing the two ATG doses, the 3-year OS, DFS, and GRFS was 69.5% vs. 63.5%, 62.2% vs. 60.3%, and 36.0% vs>. 31.7% in the 7.5 mg/kg and 10 mg/kg groups, respectively. The outcomes of OS and DFS were comparable to the above values, while GRFS had relatively distinct advantages, wherein patients with long-term disease progression-free and no severe GVHD could have a significantly improved quality of life. A retrospective study have found that cord blood transplantation without ATG has similar incidence of OS, TRM, LFS and GVHD compared with transplantation from unrelated donors, but cord blood transplantation has higher GRFS (25). This further suggests that cord blood plays a key role in our study.

Nevertheless, the present study has some limitations. This was a retrospective study, and no paired samples were available for analysis; hence, comparisons could only be made with historical data. In the next step, we plan to conduct a multicenter prospective control study to confirm the clinical results of haplo-cord-PBSCT and conduct basic studies to further explore the mechanism of UCB.

In conclusion, this study demonstrated the safety and efficacy of haplo-cord-PBSCT for hematological malignancies. The results showed that haplo-cord-PBSCT can achieve relatively rapid hematopoietic recovery and improved GRFS. Moreover, the collection of PBSCs is simpler and more acceptable to donors than BM. Therefore, we considered haplo-cord-PBSCT as an alternative transplantation protocol for the treatment of hematological malignancies. Nonetheless, these findings need to be confirmed by further prospective randomized controlled studies and in-depth studies in conjunction with disease stratification.

## Data availability statement

The raw data supporting the conclusions of this article will be made available by the authors, without undue reservation.

## Ethics statement

Written informed consent was obtained from the individual(s), and minor(s)’ legal guardian/next of kin, for the publication of any potentially identifiable images or data included in this article.

## Author Contributions

Y-JX and CZ conceived and designed the study and helped to draft the manuscript. YC,JH, YL and T-TC performed the data collection. CZ, XM, S-YW and XC performed the statistical analysis. All authors read and critically revised the manuscript for intellectual content and approved the final manuscript.

## Funding

This work was supported by the National Natural Science Foundation of China (No.81974002), Translational Research Grant of NCRCH (No. 2021WWC02).

## Acknowledgments

We thank all of the physicians, nurses for their unevaluated contribution to this study, and patients for participating in this research.

## Conflict of interest

The authors declare that the research was conducted in the absence of any commercial or financial relationships that could be construed as a potential conflict of interest.

## Publisher’s note

All claims expressed in this article are solely those of the authors and do not necessarily represent those of their affiliated organizations, or those of the publisher, the editors and the reviewers. Any product that may be evaluated in this article, or claim that may be made by its manufacturer, is not guaranteed or endorsed by the publisher.
